# Rare gastric neoplasm: Malignant glomus tumor of the stomach. A case report

**DOI:** 10.1016/j.ijscr.2021.105802

**Published:** 2021-03-23

**Authors:** Abdullah G. Alsahwan, Zainab M. Alfaraj, Jihad AlSafwani, Abdullah H. Bunaiyan, Ridha H. AlKhalifah, Sumayah A. Al-Saba'a, Sami A. Al-Momen, Qassim Aldolah

**Affiliations:** a Department of General Surgery, Qatif Central Hospital, Qatif, Saudi Arabia; b Department of Internal Medicine, Imam Abdulrahman Bin Faisal University, Dammam, Saudi Arabia; c Department of Pathology, Qatif Central Hospital, Qatif, Saudi Arabia; d Department of Internal Medicine, Qatif Central Hospital, Qatif, Saudi Arabia

**Keywords:** BP, Blood pressure, CK, Cytokeratin, CT, Computed tomography, EUS, Endoscopic ultrasound, EUS-FNB, Endoscopic ultrasound guided fine needle biopsy, GGT, Gastric glomus tumor, GT, Glomus tumor, GI, Gastrointestinal tract, GIST, Gastrointestinal stromal tumor, HPF, High power field, HR, Heart rate, IV, intravenous, LECS, laparoscopy endoscopy cooperative surgery, NSAID, Non-steroidal anti-inflammatory drug, PRBC, Packed red blood cells, SMA, smooth muscle actin, Glomus tumor “GT”, Gastric glomus tumors “GGTs”, Upper GI bleeding, Submucosal lesions

## Abstract

**Introduction:**

Glomus tumors are rare neoplasms that aris­e from neuromyoarterial canal or glomus body. They are mainly found in the peripheral soft tissue, extremities and rarely developed inside the gastrointestinal tract. In the gastrointestinal tract, the stomach is the most common site for the development of glomus tumors, and most often found in the antrum. Usually, the symptoms of gastric glomus tumors are non specific i.e (abdominal pain, GI bleeding and/or perforation) and possibly discovered incidentally during upper GI endoscopy.

**Case presentation:**

This is a-56-year-old-male, presented to the emergency department with upper GI bleeding i.e (melena), and signs of shock (HR: 110; BP:80/60), Blood tests showed Hemoglobin level: 5 g/dl. Resuscitation was started with IV fluid and transfusion of 4 units of PRBCs. After resuscitation, He gave a 10 days history of passing black tarry stool, palpitation, headache, dizziness, easily fatigability, malaise, and colicky epigastric abdominal pain. His abdomen was soft, lax with no tenderness, there was fullness at the left upper quadrant. Upper GI endoscopy was performed that showed a large gastric ulcer with adherent clots, necrotic base and oozing at the proximal part of the greater curvature, after that the bleeding was managed with a heater probe and epinephrine injections. The histopathological examination of the biopsy revealed a spindle and epithelioid tumor with the top differential diagnosis being GIST, however other submucosal lesions cannot be excluded. After that, He underwent exploratory laparotomy and wedge resection of the tumor. The final histopathology showed a malignant glomus tumor.

**Clinical discussion:**

Due to overlapping clinical and radiological features between glomus, GIST and other submucosal lesions, the histopathological examination is considered to be the gold standard for the diagnosis. Surgical resection with negative margin is the treatment of choice for gastric glomus tumors.

**Conclusion:**

Although gastric glomus tumor is a rare entity and accounts for 1% of all gastric mesenchymal tumors, it should be considered in the differential diagnosis, since preoperative biopsy is difficult and overlapping features with other submucosal lesions. Surgical treatment is the preferred option for gastric glomus tumor and long-term follow-up is required due to high metastatic and recurrence rate in the malignant type.

## Highlights

• Glomus tumors are rare neoplasms that aris­e from neuromyoarterial canal or glomus body.
• In the GI tract, stomach is the most common site for Glomus tumors.
• Symptoms usually are non specific and can be discovered incidentally during upper GI endoscopy.
• Immunohistochemistry stains after surgical excision or tissue biopsy can confirm the diagnosis.
• Surgical treatment is the preferred option for GGTs and long-term follow-up is required due to high metastatic and recurrence rate in the malignant type.

## 1 Introduction

Glomus tumors are rare neoplasms that aris­e from neuromyoarterial canal or glomus body. They are mainly found in the peripheral soft tissue, extremities and rarely developed inside the gastrointestinal tract (GIT) [1]. In the GIT, the stomach is the most common site for the development of GTs, and most often found in the antrum [2]. Usually, the symptoms of gastric glomus tumors are nonspecific i.e (abdominal pain, GI bleeding and/or perforation) and possibly discovered incidentally during upper GI endoscopy [3]. Due to overlapping clinical and radiological features between glomus, GIST and other submucosal lesions, the histopathological examination is considered to be the gold standard for the diagnosis [4]. Surgical resection with negative margin is the treatment of choice for GGTs [2]. To the best of our knowledge, this is the 12th reported case of a malignant GGT in the English language literature. We are reporting a case of a 56-year-old-male patient presented with upper GI bleeding, who was diagnosed with a malignant glomus tumor after complete surgical resection.

## 2 Case presentation

**Fig. 1:**
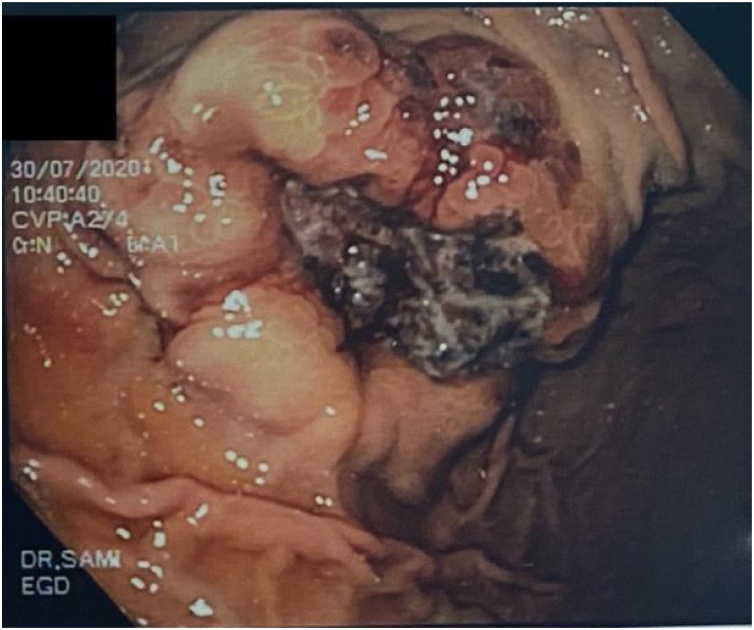
Large ulcerated mass at the greater curvature.

**Fig. 2:**
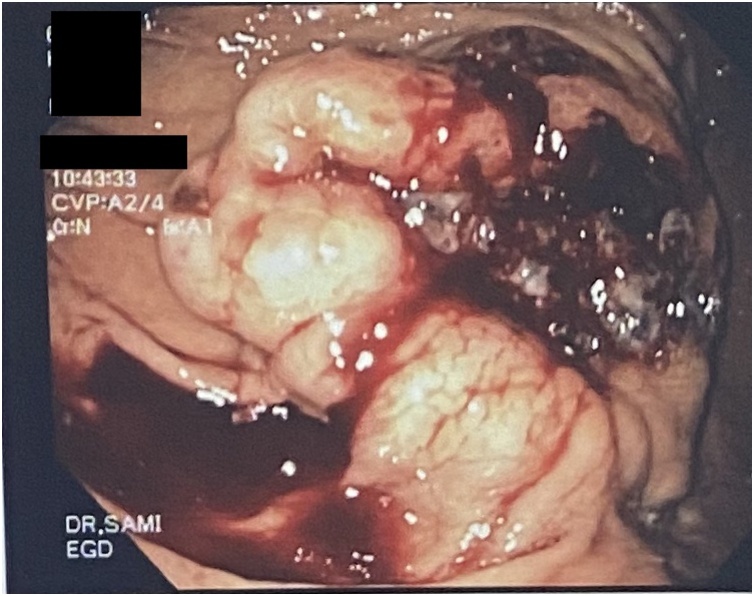
Large ulcerated mass at the  greater curvature.

**Fig. 3:**
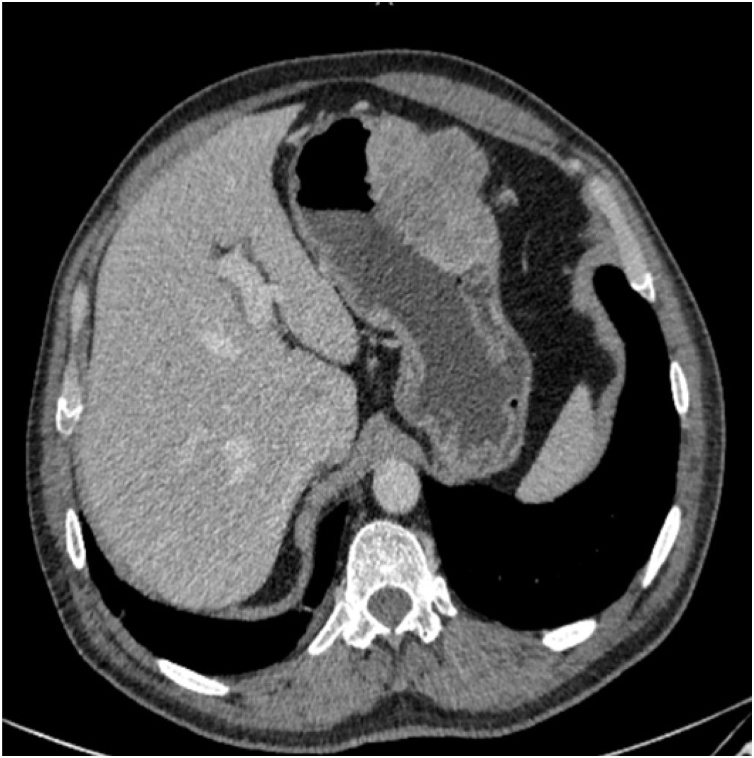
An axial plane of CT abdomen showing large lobulated mass at the greater curvature.

**Fig. 4:**
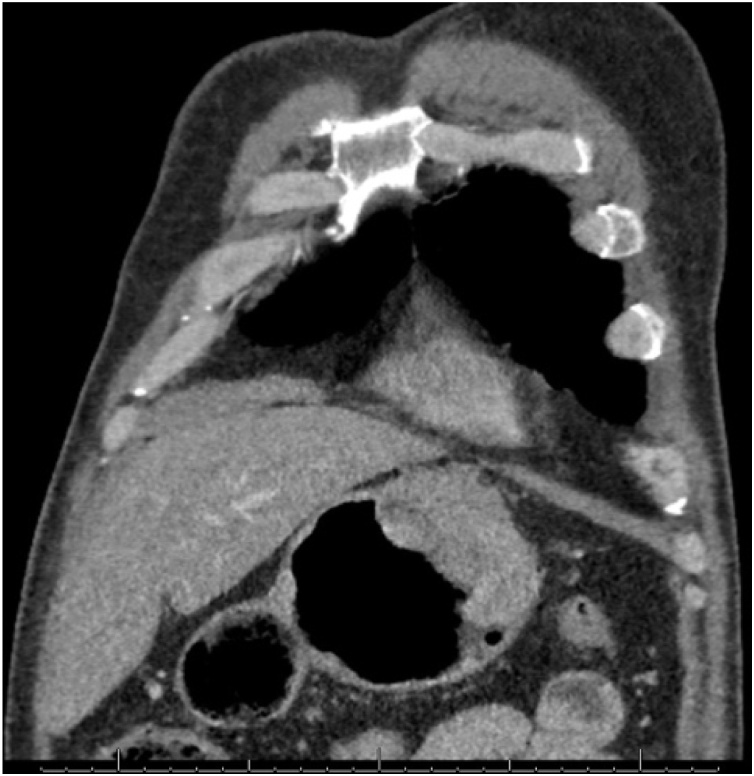
A coronal plane of CT abdomen showing large lobulated mass at the greater curvature.

**Fig. 5:**
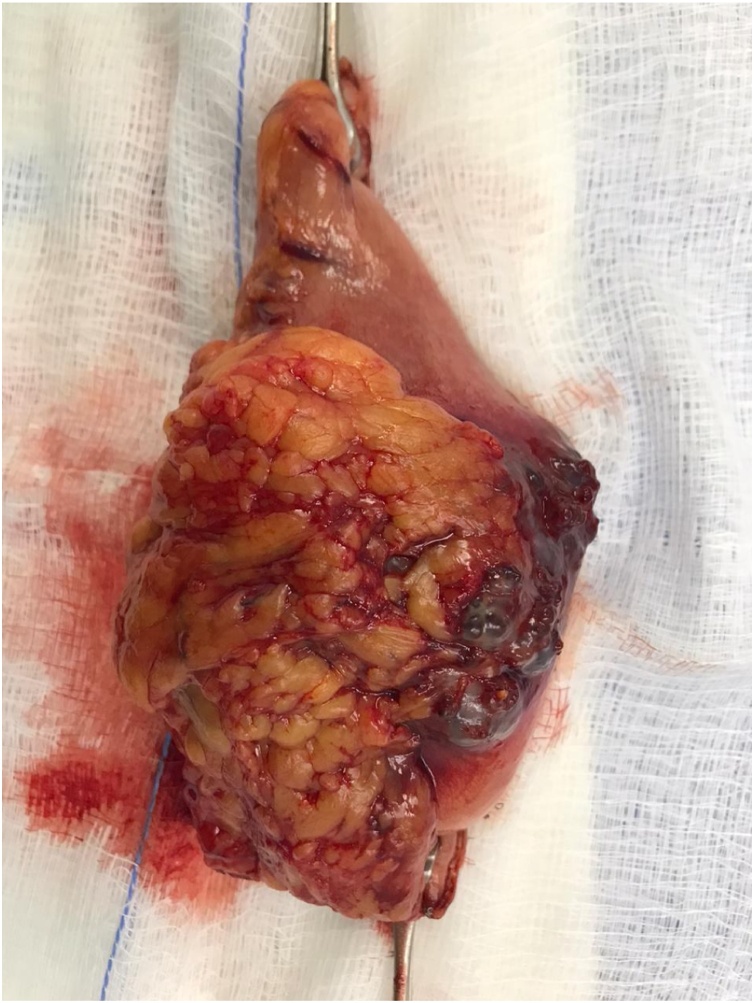
Gastric glomas tumor after wedge resection.

**Fig. 6:**
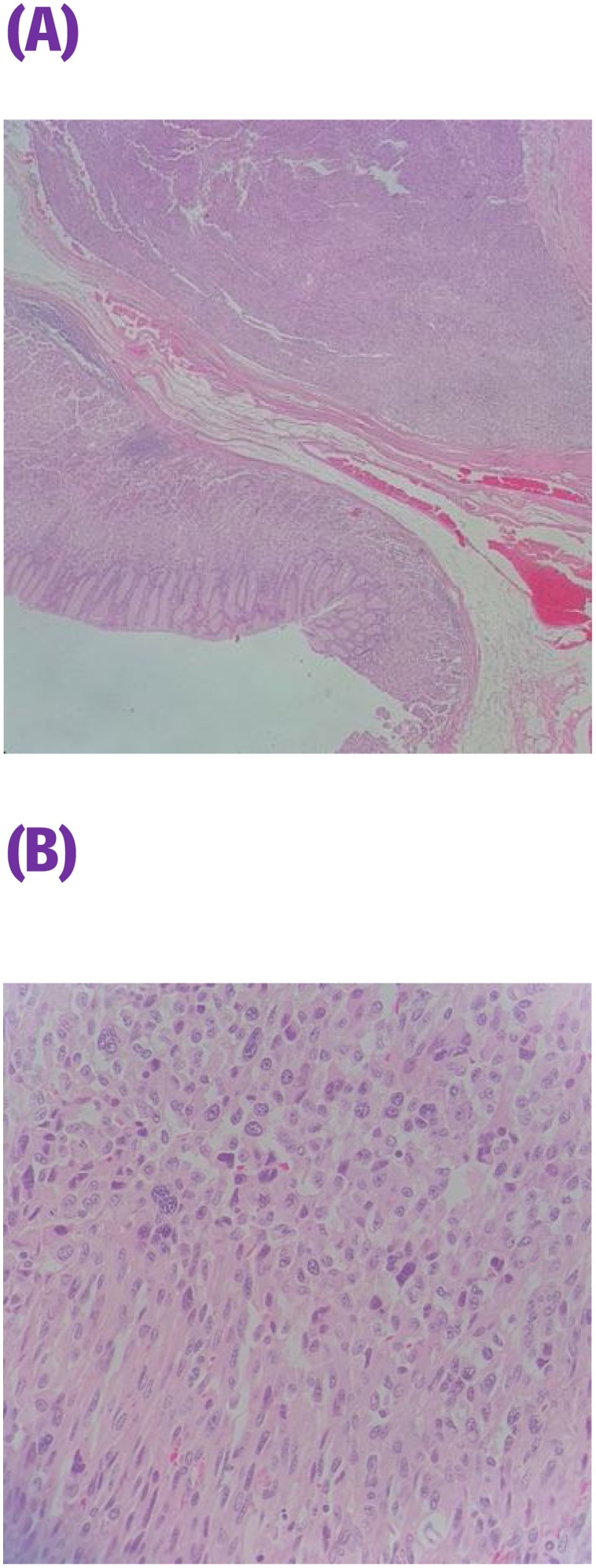
Tumor is within gastric wall composed of solid sheets of tumor cells (A) (H&E 40×). Tumor cells are epithelioid (at upper part of the picture) as well as spindled (at lower part of the picture), and featuring moderate nuclear pleomorphism (B) (H&E 400×).

**Fig. 7:**
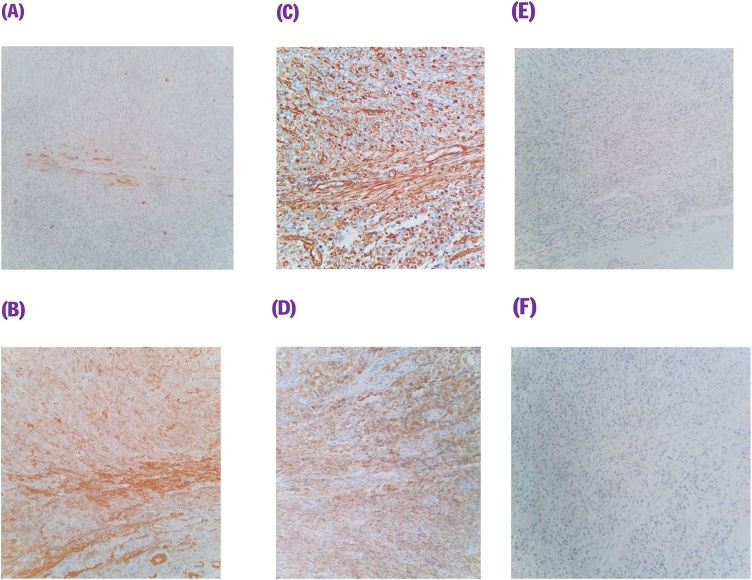
Immunohistochemical stains (200×): tumor is positive focally for (A) calponin, and diffusely for (B) smooth muscle actin, (C) vimentin, (D) synaptophysin. Tumor is negative for (E) AE1/AE3 and (F) chromogranin A.

This is a 56-year-old-male, presented to the emergency department with upper GI bleeding i.e (melena), and signs of shock (HR: 110; BP:80/60), Blood tests showed Hemoglobin level: 5 g/dl. Resuscitation was started with IV fluid and transfusion of 4 units of PRBCs. After resuscitation, He gave a 10 days history of passing black tarry stool, palpitation, headache, dizziness, easily fatigability, malaise, and colicky epigastric abdominal pain. No history of peptic ulcer disease, NSAID use, smoking or alcohol intake. His family, drug and psychological history were unremarkable. His abdomen was soft, lax with no tenderness, there was fullness at the left upper quadrant. After stabilization, upper GI endoscopy was performed that showed a large gastric ulcer with adherent clots, necrotic base and oozing at the proximal part of the greater curvature (Fig. 1, Fig. 2), after that the bleeding was managed with a heater probe, and epinephrine injections, additionally, a biopsy was taken. The histopathological examination of the biopsy revealed a spindle and epithelioid tumor with the top differential diagnosis being GIST, however other submucosal lesions cannot be excluded. Abdominal CT with IV contrast showed a large lobulated mass involving the greater curvature (Fig. 3, Fig. 4), with no local invasion or distant metastasis. On an elective basis, he underwent exploratory laparotomy that showed 8 × 5 cm, solid, oval mass at the greater curvature (Fig. 5), not invading the surrounding structures and no intra-peritoneal metastasis. So, the decision was to do a wedge resection of the tumor, which was performed by a general surgeon. Macroscopic examination revealed a 7 × 4 × 2.5 cm tumor, gray to brown in color with ulcerated surface. Multiple areas of hemorrhage were noted along with some dilated vascular spaces. Microscopic examination revealed tumor composed of nodules that consists of solid sheets of cells (Fig. 6A). The tumor cells were mainly epithelioid and round in shape with lightly pale eosinophilic cytoplasm, centrally located round or oval nuclei with delicate chromatin and inconspicuous nucleoli (Fig. 6B). Focal oncocytic, clear and spindle cells changes were seen. There was moderate nuclear pleomorphism. The mitotic rate was high, around 12 mitosis/ 10 HPF with presence of some atypical mitotic figures. There were branching and cavernous vasculature. Immunohistochemically, strong expression of smooth muscle actin (SMA) and vimentin was demonstrated along with focal expression of calponin and synaptophysin (Fig. 7). There was no immunoreaction to pan CK (AE1/AE3), chromogranin A, desmin, CD117 (C-KIT), Dog1 and S100. The Ki67 index was around 30%. This gastric tumor was diagnosed as a malignant glomus tumor. The postoperative course was uneventful, and he was discharged home in a good condition. At follow-up, 3 and 6 months after the surgery, the patient had no complaints.

## 3 Discussion

The first case report of GGT was published in 1951 by Kay et al. [5] GGT is a rare entity, it represents about 1% of all gastric mesenchymal neoplasms that arise from submucosa and muscularis propria. The peak incidence of GGTs occurs in the fifth - sixth decades of life and more commonly in females. In the stomach, most lesions occur in the gastric antrum and the size ranging between 0.8–11 cm [2,[6], [7], [8]].

Usually, these patients present with non specific symptoms i.e (abdominal pain, hematemesis, melena or perforation), or found incidentally during upper GI endoscopy, making the differentiation between submucosal gastric lesions clinically and radiologically difficult [9].

The differential diagnosis of gastric submucosal lesions apart from glomus tumor includes; GIST, leiomyoma, carcinoid tumor, hemangioma, lipoma and other rare tumors. GIST is the most common submucosal tumor in stomach. There is considerable overlapping between these submucosal tumor especially in small biopsies, thus, most cases of gastric glomus tumor were diagnosed pre-operatively as a GIST [10].

Findings that suggest the presence of gastric glomus tumor in CT with IV contrast are strong enhancement on arterial-phase and prolong enhancement on portovenous-phase, while the features in EUS are heterogeneous, hypervascular, hypoechoic masses with internal hyperechoic spots and few tubular structures mostly located on the fourth layer of the stomach wall [11,12].

Liu KL, et al., were the first authors who described the findings of GGT in MRI that include slightly hypointense on T1-weighted images, slightly hyperintense on T2-weighted images, hypervascular and prolonged enhancement after contrast administration [13].

EUS-FNB can be effective to reach the diagnosis of submucosal gastric lesions. However, this modality may fail duo to the deep location of these lesions or the sample may be insufficient to make an accurate diagnosis [9,14]. Kato et al. reported that less than 10 cases of GGTs have been diagnosed preoperatively by EUS-FNB [15].

The histopathologist faces two main challenges in diagnosing gastric glomus tumor, the first one is to differentiate glomus tumor from the exceedingly more common submucosal stromal and mesenchymal tumors, the second challenge is the categorization of this tumor into benign tumor, malignant tumor or tumor with uncertain malignant potential. Immunohistochemical stains performed on the tumor can confirm the diagnosis when the tumor stain positive for SMA, vimentin and collagen type IV and negative for CD-117 (C-KIT), DOG1, desmin, CD34, chromogranin, S-100 protein and cytokeratin [16].

Although most cases of glomus tumors are benign and have 10% recurrence rate, some malignant cases have been detected. Malignant tumors are aggressive, therefore long term follow-up is advised as the tumor may recur locally or metastasized to other organs in 40% [8,12]. After reviewing the English literature, the first case of malignant GGT was described in 1939 by Kirschbaum et al. Additionally, to date only 11 cases with malignant type were reported [8,17,18].

Folpe AL, et al., proposed the criteria for the diagnosis of malignant glomus tumor. Tumor should feature at least one of three criteria: moderate to marked nuclear atypia together with mitotic activity of more than 5 mitoses per 50 high power fields, atypical mitotic figure or size of tumor more than 2 cm with deep location [19]. Enzinger and Weiss, published the recent classification which was modified from Folpe AL, et al. In this classification, the malignant glomus tumor should feature at least one of the first two previously mentioned criteria i.e. the moderate to marked nuclear atypia together with mitotic activity of more than 5 mitoses per 50 high power fields or atypical mitotic figure. Tumor of large size and with deep location found to act mostly like a benign one. Few will ends up with distant metastasis and for that reason, these tumors are better to be categorized into glomus tumor of uncertain malignant potential [23].

The treatment of choice for GGTs is surgery. Depends on the location and the size of the tumor that play an important role in choosing the proper surgical procedure. Surgical options include open or laparoscopic Subtotal gastrectomy, or wedge resection. Complete surgical resection with negative margins “no ink on tumor” are sufficient with no need for further resection or lymph node dissection [6].

In 2008, Hiki et al., reported a case treated with laparoscopy endoscopy cooperative surgery (LECS), this option can be considered if the mass is growing in an endophytic pattern, located near the pylorus or at the gastro-esophegeal junction [20]. To date, only 3 cases were operated using this approach [21].

## 4 Conclusion

Although gastric glomus tumor is a rare entity and accounts for 1% of all gastric mesenchymal tumors, it should be considered in the differential diagnosis, since preoperative biopsy is difficult and overlapping features with other submucosal lesions. Surgical treatment is the preferred option for GGTs and long-term follow-up is required due to high metastatic and recurrence rate in the malignant type.

The work has been reported in line with the SCARE 2020 criteria [22].

## Declaration of Competing Interest

The authors report no declarations of interest.

## Sources of funding

This study did not receive any funding from governmental or private organizations.

## Ethical approval

Approval Ethical approval was obtained from the Ethical committee, Qatif Central Hospital, Qatif, Saudi Arabia. The ethical approval was signed on 01st February 2021.

## Registration of research studies

Not applicable.

## Author contribution

Abdullah G. Alsahwan: Main Author of the paper, study concept and design, data collection, data interpretation, literature review, drafting of the paper, final review of the manuscript.

Zainab M. Alfaraj, Jihad Alsafwani, Ridha H. AlKhalifah and Abdullah H Bunaiyan: Author of the paper, study concept and design, data collection, data interpretation, literature review, drafting of the paper, final review of the manuscript.

Qassim Aldolah and Sami Almomen: Supervisor; Treating physicians of the patient, study concept and design, data collection, data interpretation, literature review, drafting of the paper, final review of the manuscript.

Sumayah A. Al-Saba'a: provide the histopathological part.

## Guarantor

Dr. Abdullah Ghazi AlSahwan.

## Provenance and peer review

Not commissioned, externally peer-review.
